# Simulation of interfacial debonding in hollow particle reinforced composites with VCFEM

**DOI:** 10.1038/s41598-024-57451-x

**Published:** 2024-03-22

**Authors:** Zhiyi Wang, Rui Zhang

**Affiliations:** Engineering Mechanics Department of Kunming, University of Science and Technology, Kunming, 650500 China

**Keywords:** Mechanical engineering, Composites, Mechanical properties, Computational science

## Abstract

The addition of hollow glass microsphere into composites is a method to improve mechanical properties. However, the interfacial debonding of hollow microsphere inevitably causes a decrease in the mechanical properties of the material, which ultimately leads to the failure of the composites. In the numerical simulation of such hollow particle-reinforced composites, the ordinary displacement finite element requires a large number of meshes, which undoubtedly greatly increases the computational cost. In this paper, a new VCFEM is proposed to solve this problem by establishing a two-dimensional Voronoi cell finite element model, deriving the residual energy generalized function of hollow particle-reinforced composites, and calculating the interface debonding. The simulation results are compared with the commercial software MARC, ABAQUS to verify the effectiveness of this VCFEM. The results show that this VCFEM greatly improves the computational efficiency while ensuring the accuracy. Based on this model, this paper also investigates the effect of the generation of interfacial debonding on the overall structure and the effect of different wall thickness of hollow particles on the damage of element debonding.

## Introduction

Hollow particle-reinforced composites have been widely utilized across various industries, such as aviation, military, nuclear power, and construction. Hollow Glass Microspheres (HGM) are characterized by their hollow structure, lightweight, wear resistance, and corrosion resistance. Research indicates that the addition of HGM to composites can enhance their mechanical properties. Swetha, C.^1^ conducted a series of quasi-static uniaxial compression experiments to explore the influence of hollow microsphere content and wall thickness on the mechanical characteristics of foams. Scotta, R.^2^ investigated the impact of glass microspheres on weight reduction properties and compressive strength of glass microsphere reinforced cementitious composites under quasi-static and high strain rate conditions. Blanco, F.^3^ demonstrated that the compressive strength of lightweight cementitious materials surpassed that of conventional perlite lightweight cement upon the incorporation of hollow microspheres.

Tiwari, Vikrant^4^ showed that the addition of hollow glass microsphere to the cement matrix significantly improved the acoustic insulation properties of the material. In the investigation of hollow glass microsphere reinforced matrix composites, numerous studies have assessed the mechanical properties by examining the microstructure-property relationship^5–8^. It has been observed that Eshelby's solution, which is based on the assumption of spatially uniform strain within an elliptical homogeneous envelope, does not accurately predict the elastic properties of the material. Consequently, researchers have incorporated considerations for the non-uniformity of the microstructure to develop more precise mathematical models^9–11^. However, challenges such as hollow microsphere aggregation, microsphere cracking, and interface debonding contribute to the deterioration of mechanical properties^12–14^, resulting in failure damage of the composites and hindering the model's predictive accuracy. Therefore, in the exploration of hollow glass microsphere reinforced matrix composites, a comprehensive understanding of the influence of damage on the material's mechanical properties is crucial. Given the stress concentration at damaged interfaces and the presence of numerous randomly distributed inclusions of varying sizes and shapes in the composites, conventional displacement-based finite element methods struggle to simulate the complexity of such composites. Consequently, specialized finite element methods have been developed to mitigate computational costs.

The VCFEM (Voronoi Cell Finite Element Method) is a convenient approach that can greatly enhance computational efficiency. In comparison to traditional finite element models, VCFEM is able to reduce the degrees of freedom effectively, leading to a significant improvement in computational productivity. Zhang^15^ introduced a hybrid finite element method for analyzing the mechanical response of two-dimensional heterogeneous materials containing randomly dispersed rigid inclusions of arbitrary sizes. Ghosh and Moorthy proposed the Voronoi cell finite element method^16–18^, which has been utilized as a precise and efficient tool for modeling non-uniform microstructures with diverse shapes, sizes, and dispersed heterogeneities. Guo^19^ suggested the use of Voronoi cells to simulate creep, thermal, and plastic strains in two-dimensional particle-reinforced composites. Zhang^20^ developed a new finite element model, MHSFEM, that simplifies mesh generation and, when combined with VCFEM, enables the analysis of unique microstructures, expanding the application scope of VCFEM. Zhang^21^ proposed the SVCFEM to simulate stress concentration at crack tips in homogeneous materials. Ghosh^22^ employed VCFEM to study interfacial debonding in fiber-reinforced composites, where the matrix and inclusions are connected through flexible elements known as springs. In contrast, this paper uses shared node pairs to simulate interface debonding. Li and Ghosh^23,24^ introduced the XVCFEM to accurately model the propagation of multiple cracks in homogeneous materials. Zhang^21^ utilized VCFEM to analyze multi-cracking in homogeneous materials. These methods focus on simulating crack propagation in homogeneous materials. The current study proposes a new VCFEM to simulate interface debonding in hollow particle-reinforced composites. Zhang^25^ extended the Voronoi cell finite element method to incorporate arbitrarily distributed inclusion reinforcements with homogeneous phases in composites. Han^26^ developed two new elements based on the Voronoi cell finite element model and conducted fracture simulations of materials with large voids under internal pressure. Hao^27^ established a two-dimensional Voronoi cell finite element model to analyze the fatigue behavior of homogeneous materials with dispersed voids in the matrix. Rao^28^ proposed a two-dimensional VCFEM, formulated with plastic, thermal, and creep strain. In conclusion, VCFEM is well-suited for simulating composites with numerous inclusions. Currently, VCFEM is predominantly used for micromechanical damage analysis, such as crack propagation in homogeneous materials, without considering the impact of hollow particle-reinforced composites in crack propagation and interfacial debonding.

Therefore, the objective of this study is to enhance the existing advanced VCFEM to investigate the damage behavior occurring at the interface between the matrix and inclusions in hollow particle-reinforced composites. A novel VCFEM approach is proposed based on a hybrid stress model to characterize the two-phase damage behavior in such composites. A unique cell containing a hollow inclusion is defined based on the principle of complementary residual energy, where stress and displacement are treated as independent fields. The stress field is assumed to satisfy equilibrium equations in advance, represented by a polynomial stress function combined with the inverse stress function within the matrix. A correction function is formulated by introducing Lagrange multipliers into the function. By establishing a relationship between the stress function and the displacement variable, the equations are transformed into a solvable form, and the resulting model is presented following the standard FEM solution format. A new remeshing strategy is implemented to investigate the issue of interfacial debonding in hollow particle-reinforced composites through numerical examples, demonstrating the feasibility of this novel VCFEM approach.

## Hollow particle debonding Voronoi cell construction

**Figure 1:**
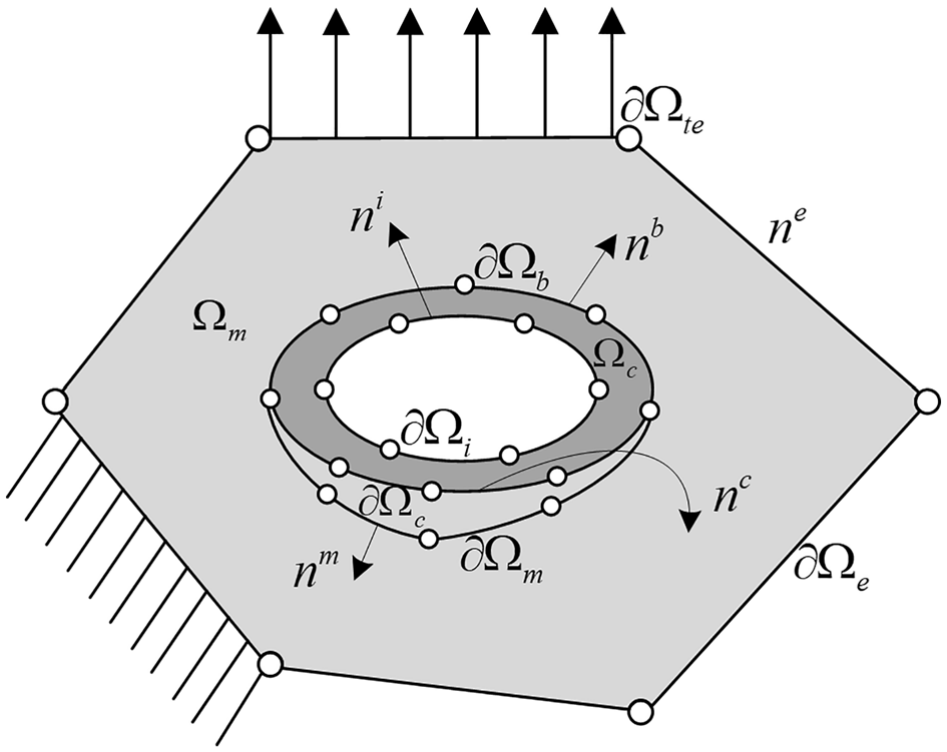
Voronoi cell model with a hollow inclusion, considering interfacial debonding.

In the following, the two-dimensional linear elasticity problem is mainly investigated and numerical examples are carried out under plane stress. In order to analyze the effect of debonding between inclusion and matrix on the microevolution of the structure and the macroscopic mechanical properties, we consider a cell model as in Fig. 1. The inclusion and matrix phases of the hollow particle debonding Voronoi cell are denoted as $\Omega_{c}^{{}}$, $\Omega_{m}^{{}}$_._ $n^{e}$ is the direction of the outer normal to the cell boundary $\partial \Omega_{e}^{{}}$. $n^{b}$ is the direction of the outer normal to the perfectly bonded matrix-inclusion interface $\partial \Omega_{b}^{{}}$. $n^{i}$ is the direction of the outer normal to the hollow inclusion interface $\partial \Omega_{i}^{{}}$. $n^{m}$ and $n^{c}$ are the directions of the outer normal at the debonding matrix interface $\partial \Omega_{m}^{{}}$ and the debonding inclusion interface $\partial \Omega_{c}^{{}}$, respectively.

At the bonding interface $\partial \Omega_{b}^{{}}$ between the inclusion and the matrix, the surface force continuity condition is satisfied.

1 $$n^{b} \cdot \left( {\sigma^{m} - \sigma^{c} } \right) = 0$$

At the debonding interface $\partial \Omega_{m}^{{}}$ and $\partial \Omega_{c}^{{}}$, the surface force is zero.

2 $$n^{m} \cdot \sigma^{m} = 0$$

3 $$n^{c} \cdot \sigma^{c} = 0$$

At the interface $\partial \Omega_{i}^{{}}$ between inclusion and hollow part, the surface force is zero.

4 $$n^{i} \cdot \sigma^{c} = 0$$

The modified residual energy generalized function is obtained by applying Lagrange multipliers to apply constraints based on the stress hybrid element generalized function formulation:

5 $$\begin{gathered} \Pi_{mc}^{e} = \sum\limits_{e} {} \left\{ {\int_{{\Omega_{m}^{{}} }} \frac{1}{2} \sigma_{{}}^{m} :S:\sigma_{{}}^{m} d\Omega + \int_{{\Omega_{c}^{{}} }} \frac{1}{2} \sigma_{{}}^{c} :S:\sigma_{{}}^{c} d\Omega + \int_{{\partial \Omega_{te}^{{}} }} {\overline{T} \cdot ud\partial \Omega - \int_{{\partial \Omega_{e}^{{}} }} {n^{e} \cdot \sigma^{m} \cdot u^{e} d\partial \Omega } } } \right. \hfill \\ + \int_{{\partial \Omega_{b}^{{}} }} {n^{b} \cdot \left( {\sigma_{{}}^{m} - \sigma_{{}}^{c} } \right) \cdot u^{b} d\partial \Omega - \int_{{\partial \Omega_{m}^{{}} }} {n^{m} \cdot \sigma^{m} \cdot u^{m} d\partial \Omega } } \hfill \\ \left. { - \int_{{\partial \Omega_{c}^{{}} }} {n^{c} \cdot \sigma^{c} \cdot u^{c} d\partial \Omega + \int_{{\partial \Omega_{i}^{{}} }} {n^{i} \cdot \sigma^{c} \cdot u^{i} d\partial \Omega } } } \right\} \hfill \\ \end{gathered}$$

where $u^{m}$ and $u^{c}$ denote the debonding crack displacements and the cell boundary displacement field is obtained from a linear interpolation function.

Expression of residual energy functional after finite element discretization:

6 $$\Pi_{mc}^{e} = \sum\limits_{e} {\left( {\frac{1}{2}\beta_{m}^{T} H_{{}}^{m} \beta_{m}^{{}} + \frac{1}{2}\beta_{c}^{T} H_{{}}^{c} \beta_{c}^{{}} - \beta_{m}^{T} G_{e}^{{}} q^{e} - \beta_{c}^{T} G_{cb}^{{}} q^{b} - \beta_{m}^{T} G_{mm}^{{}} q^{m} - \beta_{c}^{T} G_{cc}^{{}} q^{c} + \beta_{c}^{T} G_{ci}^{{}} q^{i} + \overline{f} q_{{}}^{e} } \right)}$$

In the above equation, the matrix $H^{m} ,H^{c} ,G_{e}^{{}} ,G_{mb}^{{}} ,G_{cc}^{{}} ,G_{cb}^{{}} ,G_{mm}^{{}} ,G_{ci}^{{}}$ is defined as:

7 $$H^{m} = \int_{{\Omega_{m}^{{}} }} {P_{{}}^{mT} S_{m}^{{}} P^{m} d\Omega } ,H^{c} = \int_{{\Omega_{c}^{{}} }} {P_{{}}^{cT} S_{c}^{{}} P^{c} d\Omega }$$

8 $$G_{e}^{{}} = \int_{{\partial \Omega_{e}^{{}} }} {P^{mT} n^{eT} Ld\partial \Omega } ,G_{mb}^{{}} = \int_{{\partial \Omega_{b}^{{}} }} {P^{mT} n^{bT} L^{b} d\partial \Omega }$$

9 $$G_{cc}^{{}} = \int_{{\partial \Omega_{c}^{{}} }} {P^{cT} n^{cT} L^{c} d\partial \Omega } ,G_{cb}^{{}} = \int_{{\partial \Omega_{b}^{{}} }} {P^{cT} n^{bT} L^{b} d\partial \Omega }$$

10 $$G_{mm}^{{}} = \int_{{\partial \Omega_{m}^{{}} }} {P^{mT} n^{mT} L^{m} d\partial \Omega } ,G_{ci}^{{}} = \int_{{\partial \Omega_{i}^{{}} }} {P^{cT} n^{iT} L^{i} d\partial \Omega }$$

The identification of Gaussian points in the integral computation is realized by dividing multiple quadrangles in the matrix of each phase.$P^{m} ,P^{c}$ is the known stress function at the relative point locations of the matrix, inclusion.

By deriving $\Pi_{mc}^{e}$ from the stress function $\beta_{m}^{{}} ,\beta_{c}^{{}}$, we obtain:

11 $$\frac{{\partial \Pi_{mc}^{e} }}{{\partial \beta_{m}^{{}} }} = 0,\frac{{\partial \Pi_{mc}^{e} }}{{\partial \beta_{c}^{{}} }} = 0$$

A weak expression for the relationship between the motions in the element can be obtained:

12 $$\begin{gathered} H^{m} \beta_{m}^{{}} = G_{e}^{{}} q^{e} - G_{mb}^{{}} q^{b} + G_{mm}^{{}} q^{m} \hfill \\ H^{c} \beta_{c}^{{}} = G_{cb}^{{}} q^{b} + G_{cc}^{{}} q_{{}}^{c} - G_{ci}^{{}} q^{i} \hfill \\ \end{gathered}$$

13 $$\left( {\begin{array}{*{20}c} {H_{m}^{{}} } & 0 \\ 0 & {H_{c}^{{}} } \\ \end{array} } \right)\left( {\begin{array}{*{20}c} {\beta_{m}^{{}} } \\ {\beta_{c}^{{}} } \\ \end{array} } \right) = \left( {\begin{array}{*{20}c} {G_{e}^{{}} } & { - G_{mb}^{{}} } & {G_{mm}^{{}} } & 0 & 0 \\ 0 & {G_{cb}^{{}} } & 0 & {G_{cc}^{{}} } & { - G_{ci}^{{}} } \\ \end{array} } \right)\left( {\begin{array}{*{20}c} {q^{e} } \\ {q^{b} } \\ {q^{m} } \\ {q^{c} } \\ {q^{i} } \\ \end{array} } \right)$$

By taking the derivative of $\Pi_{mc}^{e}$ with respect to $q^{e} ,q^{b} ,q^{m} ,q^{m} ,q^{i}$, we obtain:

14 $$\frac{{\partial \Pi_{mc}^{e} }}{{\partial q_{{}}^{e} }} = 0,\frac{{\partial \Pi_{mc}^{e} }}{{\partial q_{{}}^{b} }} = 0,\frac{{\partial \Pi_{mc}^{e} }}{{\partial q_{{}}^{m} }} = 0,\frac{{\partial \Pi_{mc}^{e} }}{{\partial q_{{}}^{c} }} = 0,\frac{{\partial \Pi_{mc}^{e} }}{{\partial q_{{}}^{i} }} = 0$$

A weak expression for the surface force boundary condition can be obtained:

15 $$\sum\limits_{e} {\left( {\begin{array}{*{20}c} {G_{e}^{{}} } & { - G_{mb}^{{}} } & {G_{mm}^{{}} } & 0 & 0 \\ 0 & {G_{cb}^{{}} } & 0 & {G_{cc}^{{}} } & { - G_{ci}^{{}} } \\ \end{array} } \right)^{T} } \left( {\begin{array}{*{20}c} {\beta_{m}^{{}} } \\ {\beta_{c}^{{}} } \\ \end{array} } \right) = \left( {\begin{array}{*{20}c} {\overline{F}^{e} } \\ 0 \\ 0 \\ 0 \\ 0 \\ \end{array} } \right)$$

Among them,

16 $$\overline{F}^{eT} = \int_{{\partial \Omega_{tc}^{{}} }} {\overline{f} }^{T} Ld\partial \Omega$$

Coefficient $\beta$ can be written:

17 $$\beta = H^{ - 1} Gq$$

Substituting Eq. (17) into Eq. (15) yields

18 $$\sum\limits_{e} {G^{T} H^{ - 1} Gq = \sum\limits_{e} {\overline{F} } }$$

19 $$\sum\limits_{e} {K_{e}^{{}} q = \sum\limits_{e} {\overline{F} } }$$

## A strategy for matrix-inclusion interface debonding remeshing

In order to realistically simulate the interfacial debonding process of hollow particle reinforced composites, with the change of the debonding region, it is inevitable to change the corresponding mesh structure, so the matrix-inclusion interface remeshing strategy is used to simulate the gradual debonding of the matrix-inclusion interface. Since we mainly study the finite element method, we could have used this very simple and effective strategy for modeling the interface debonding.

**Figure 2:**
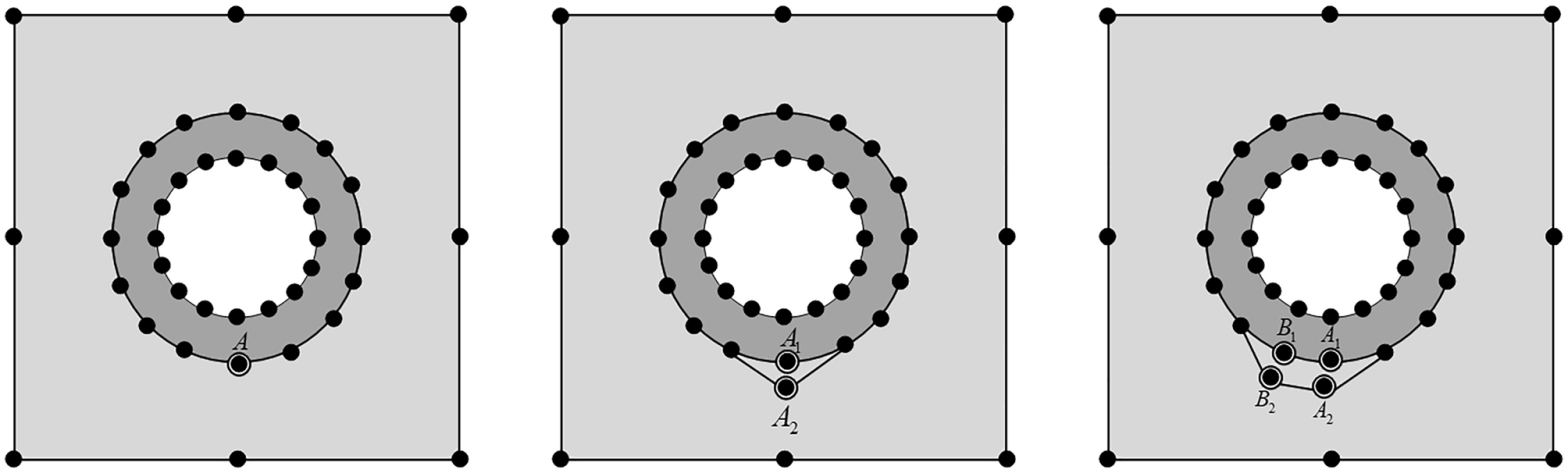
Mesh remeshing steps for debonding at the matrix-inclusion interface.

Assuming that a particle reinforced composite has produced a section of debonding between the interfaces of matrix and inclusion in an element at the beginning of an incremental step during a certain loading process, according to certain fracture mechanics principles, when the stress at a node reaches or exceeds a critical state, the initial crack tip produces an extension forward at that node extending the interfaces of the matrix and the inclusion. In the first step, the stress state of all the nodes of the interface is determined, and if the node stress reaches or exceeds the critical stress, the interface produces debonding. A second step is connecting the two nodes of the debonding interface to generate a new debonding layer. By the example of A in Fig. 2, when A reaches the critical stress, debonding occurs at the interface. Since the matrix and the inclusion share the same node at point A before the new debonding occurs, this is not a problem in the bonding state. However, with the emergence of the new debonding, the displacement of the part of the matrix at node A and the part of the inclusion at node A are no longer identical. In order to ensure that the crack surface force is zero, we use the node pair A1 and A2 to represent the point A on the inclusion and the point A on the matrix, respectively. The corresponding node variables satisfy the following relationship:

20 $$\Delta u_{{A_{1}^{{}} }}^{{}} = \Delta u_{{A_{2}^{{}} }}^{{}} = \Delta u_{{A_{{}}^{{}} }}^{{}}$$

When the original crack tip A extends to point B, if the stress state at node B reaches or exceeds the critical state, then the debonding continues at point B, producing a new debonding layer, and the next step continues to judge whether the stress state at the other nodes reaches the critical state.

## Numerical examples

There are two types of numerical examples in this section: The first type of example is to verify the validity of this VCFEM. The validity is verified by comparing the results with those of the commercial computational software MRAC and ABAQUS. The second type of example is to use this element to simulate a composite material containing a large number of randomly distributed hollow inclusions and to calculate the gradual cracking process at the interface between the matrix and the hollow inclusions under load. Its validity is also verified by comparing certain states of the cracking process with simulation analysis using MARC software.

The same number of polynomials and stress function terms are used in all the calculations.

### A matrix-inclusion element

**Figure 3:**
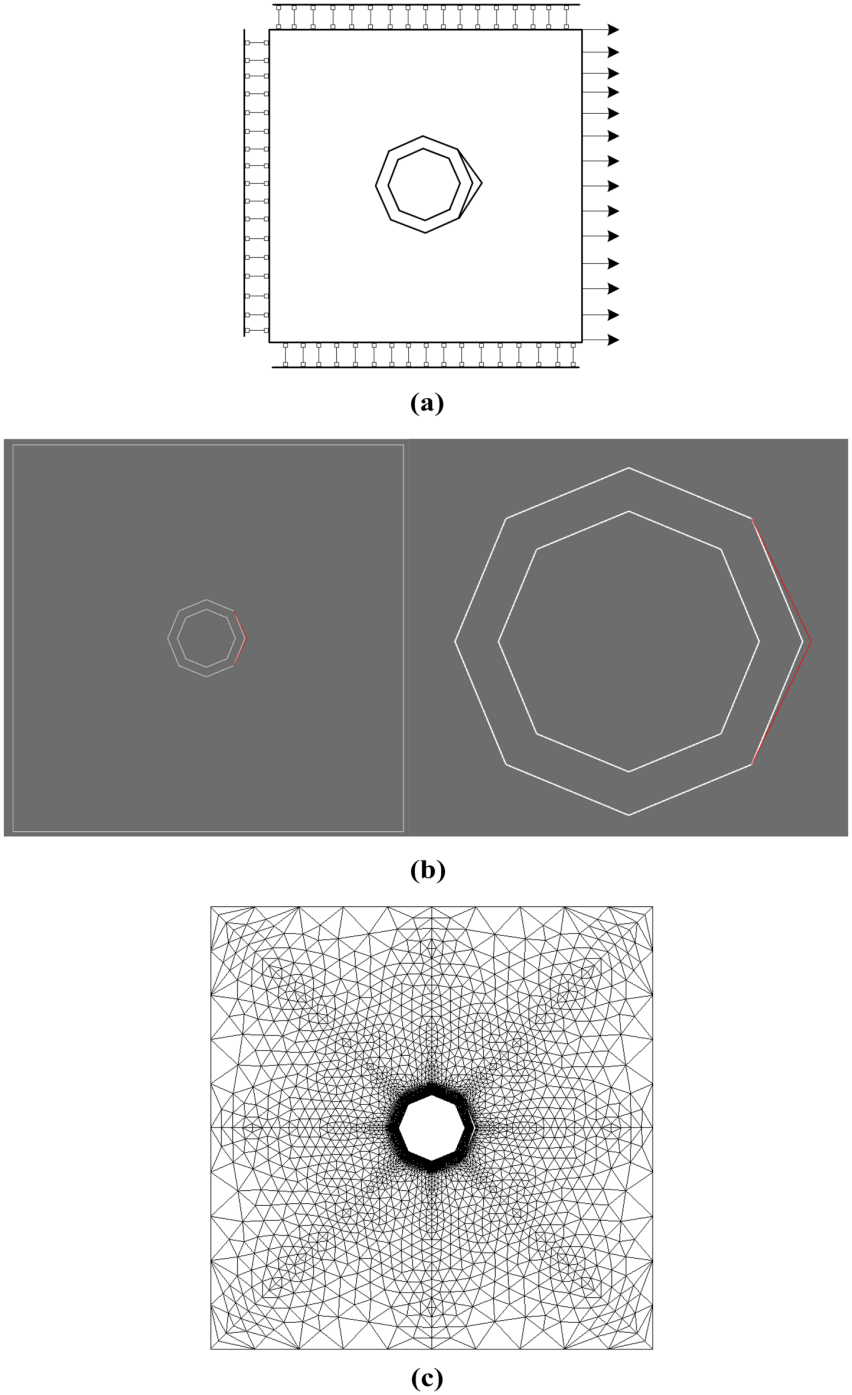
(**a**) Model structural working conditions, (**b**) VCFEM mesh structure model and (**c**) MARC mesh structure model.

In this example, the VCFEM model consists of a simple Voronoi element, the MARC and ABAQUS models consist of a fine finite element mesh with a large number of elements. The validity of the VCFEM is verified by comparing it with the results of MARC and ABAQUS. The model to be simulated is an $2 \times 2mm^{2}$ square matrix containing a hollow inclusion with a radius of $0.2mm^{{}}$ and the hollow part of the inclusion with a radius of $0.15mm^{{}}$. The inclusion is located at the center of the square matrix. The inclusion is represented by an octagonal structure in which the right two sides are cracked. For comparative analysis, the VCFEM model, MARC model and ABAQUS model are subjected to exactly the same boundary conditions and displacement loads are shown in Fig. 3a, the vertical displacement on the top boundary is 0, the vertical displacement on the bottom boundary is 0, and the horizontal displacement on the left boundary is 0, and the horizontal displacement on the right boundary is ${0}{\text{.0002}}\;mm$.

The MARC model consists of 9856 second-order plane stress triangular elements, with 5107 nodes as shown in Fig. 3c; the ABAQUS model consists of 3337 CPS4R elements and 100 CPS3 elements, with 3509 nodes; the VCFEM model consists of a single Voronoi element as shown in Fig. 3b, with stress functions in both the matrix and Voronoi elements containing 99 terms, composed of 63 polynomial stress functions and 33 interaction stress function terms. The material properties used in the modeling analysis are as follows:

Inclusion:$$E_{c}^{{}} = 80000\;MPa,\;\upsilon_{c}^{{}} = 0.25,\;r_{c}^{{}} = 0.2\;mm,\;r_{h}^{{}} = 0.15\;mm$$

Matrix:$$E_{m}^{{}} = 40000\;MPa,\;\upsilon_{{\text{m}}}^{{}} = 0.2$$

**Figure 4:**
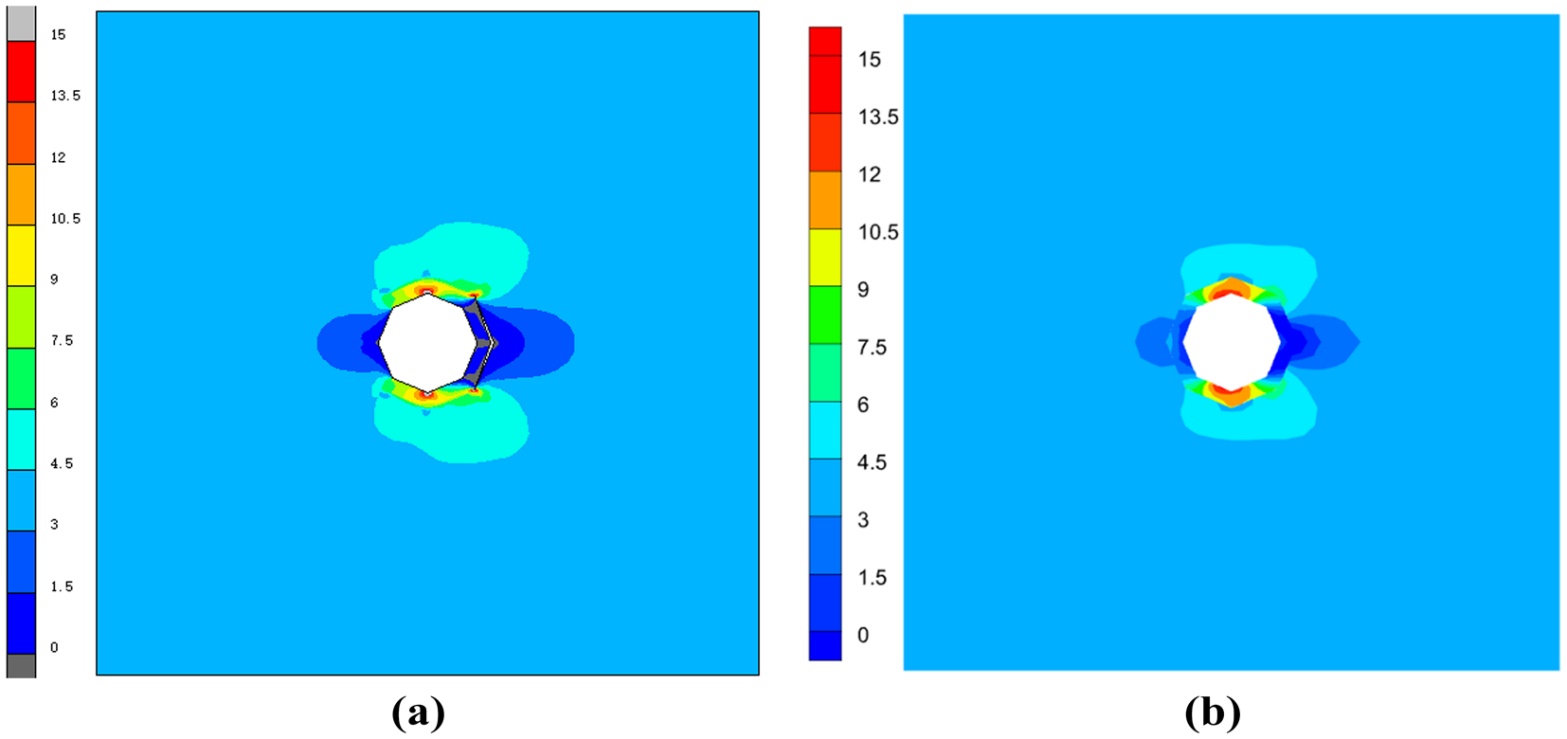
Horizontal stress $\sigma_{x}^{{}}$ cloud plots of (**a**) MARC model and (**b**) VCFEM model.

**Figure 5:**
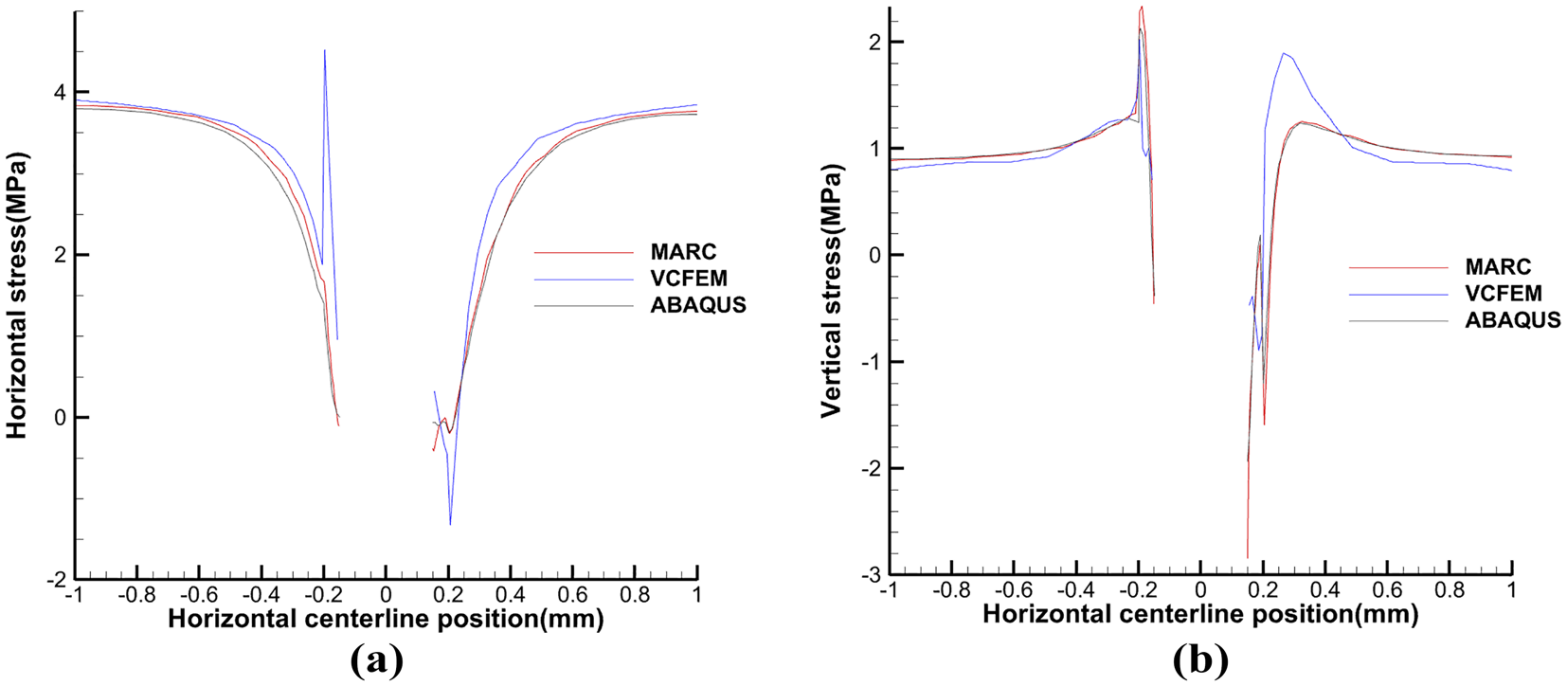
Distribution of stress (**a**) $\sigma_{x}^{{}}$, (**b**) $\sigma_{y}^{{}}$ on the horizontal centerline in MARC, VCFEM and ABAQUS models.

**Figure 6:**
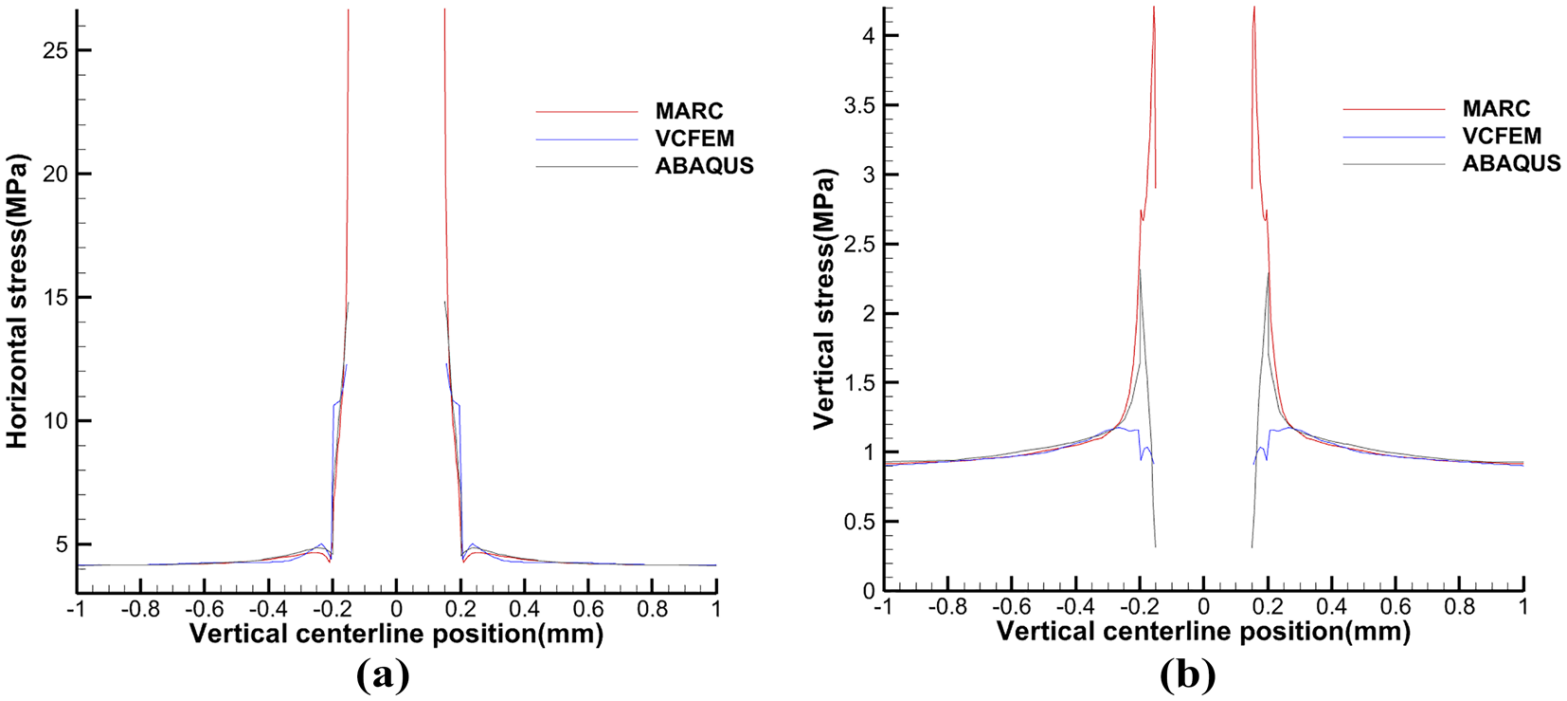
Distribution of stress (**a**) $\sigma_{x}^{{}}$, (**b**) $\sigma_{y}^{{}}$ on the vertical centerline in MARC, VCFEM and ABAQUS models.

The critical normal stress at the interface is 1.05 N and the critical load is 0.0002 m. Comparative analysis of the results in Fig. 4 shows that the three models can be in good agreement with each other. From Figs. 5 and 6 at the centerline position of VCFEM, MARC, and ABAQUS $\sigma_{x}^{{}}$,$\sigma_{y}^{{}}$ comparison, it can be seen that there are different peaks at the matrix-inclusion interface and at the crack, and there are different degrees of stress concentration in the hollow part of the inclusion, and the stress decreases rapidly to the minimum value at the crack. The stress components of the three models are more or less different except at the interface of matrix and inclusion, which is mainly caused by these two reasons: (I) in VCFEM, the matrix part and the inclusion part within each element are assumed to be the stress function, so that describing the stress field with two continuous functions that satisfy the perfect continuity at all the interfaces of the matrix and the inclusion is difficult; (II) in the MARC model, there is only one node at the same location on the interface of matrix and inclusion, and there is only one value of the stress component at the interface node obtained by the software, which does not reflect the stress discontinuity applied at the interface. Whereas, in the VCFEM model and ABAQUS, there are two values of stress at the interface point.

### Interfacial debonding of a matrix-inclusion element

In this example, the VCFEM model consists of a simple Voronoi element and the MARC and ABAQUS models consist of a fine finite element mesh with a large number of elements. The validity of the VCFEM is verified by comparing it with the results of MARC and ABAQUS. The model to be simulated is an $2 \times 2\;mm^{2}$ square matrix containing a hollow inclusion with a radius of $0.2\;mm^{{}}$ and the hollow part of the inclusion with a radius of $0.15\;mm^{{}}$. The inclusion is located in the center of the square substrate and inclusion interface consisted of 16 straight edge segments. For comparative analysis, the VCFEM model, MARC model and ABAQUS model are subjected to exactly the same boundary conditions and displacement loads, the vertical displacement on the top boundary is 0, the vertical displacement on the bottom boundary is 0, and the horizontal displacement on the left boundary is 0, and the horizontal displacement on the right boundary is ${0}{\text{.0002}}\;mm$.Initially, each side does not contain debonding, and as the load increases, debonding of different numbers of sides occurs when the stress state at each node of the inclusion reaches or exceeds the critical stress state.

In order to correspond to the debonding process, four MARC models were established for comparative analysis, including 13424 second-order isotropic plane stress triangle elements (containing 6970 nodes); an ABAQUS model consisting of 8316 CPS4R elements and 246 CPS3 elements (containing 8562 nodes); and a VCFEM model consisting of a Voronoi element. Both the matrix and Voronoi elements contain stress functions with 99 terms, composed of 63 polynomial stress function terms and 33 interaction stress function terms. The material properties of the model are the same as above.

**Figure 7:**
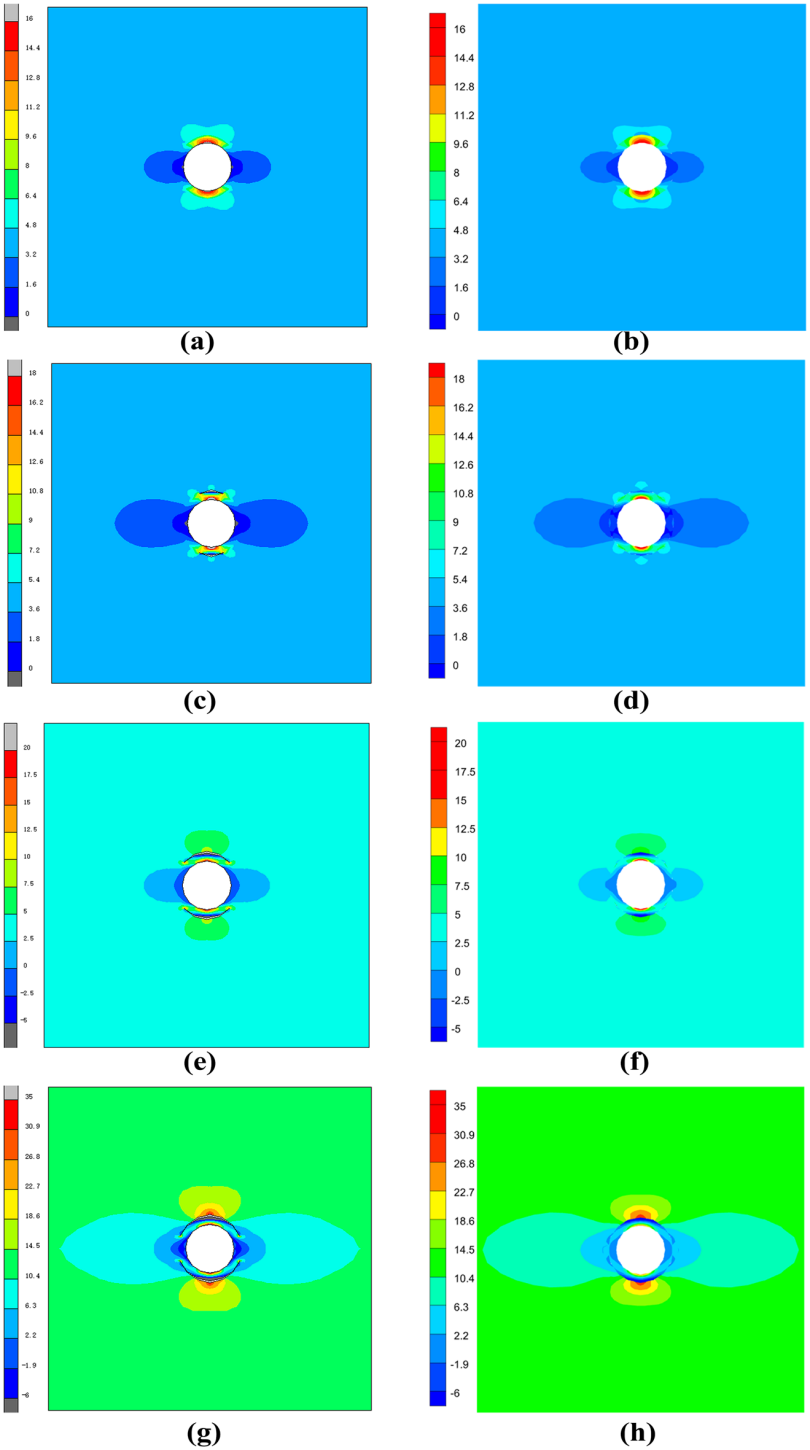
Horizontal stress cloud plots between (**a**,**c**,**e**,**g**) MARC model and (**b**,**d**,**f**,**h**) VCFEM model.

**Figure 8:**
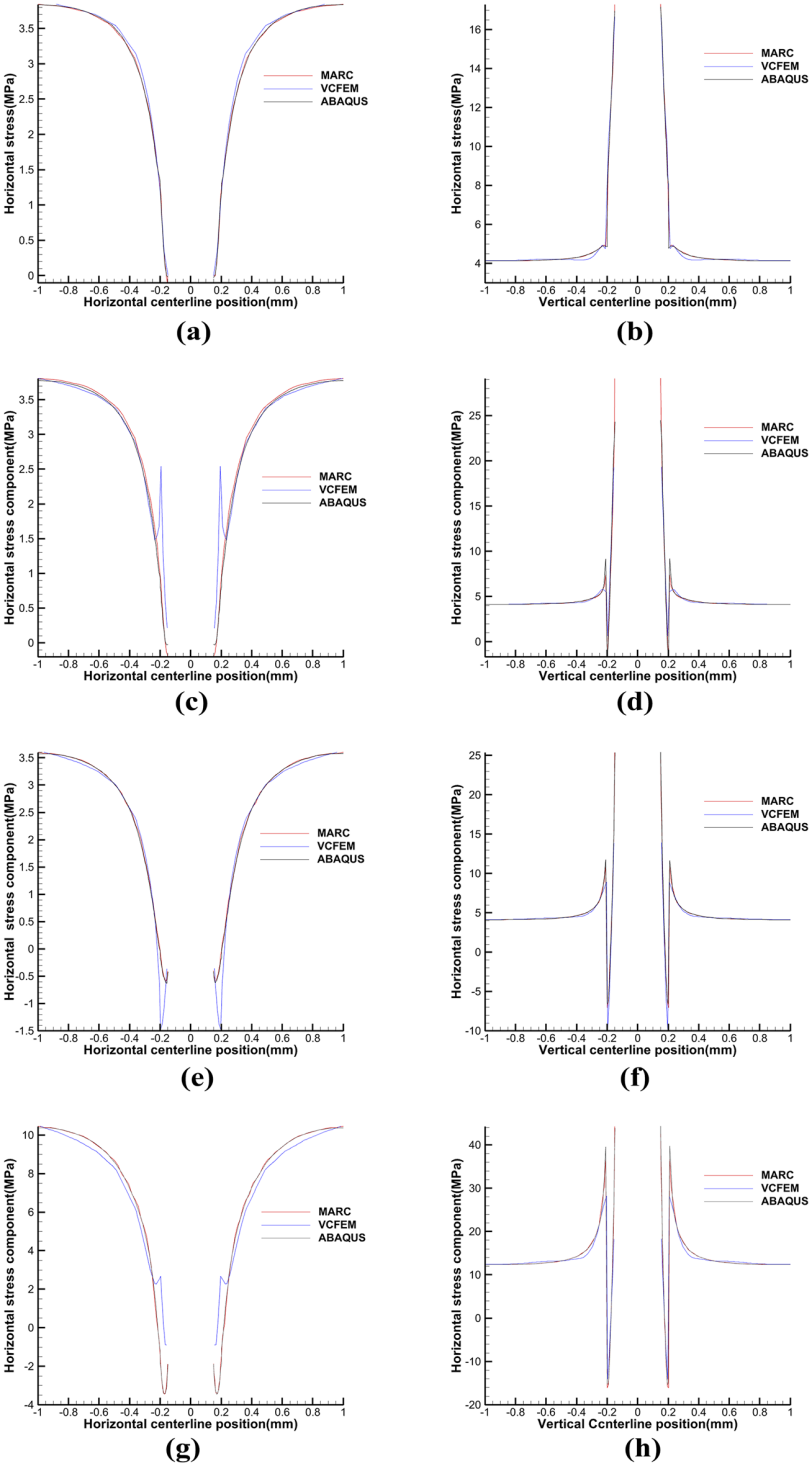
Distribution of horizontal stress on the horizontal centerline and vertical centerline in MARC,VCFEM and ABAQUS models.

The critical normal stress at the interface was 2.31N, and the critical displacement loads for debonding with different numbers of sides were 0.0002mm, 0.00045mm, and 0.00125mm, respectively. Figure 7 gives the stress cloud plots for MARC and VCFEM at complete bonding, 2 × 2 debonded sides, 2 × 4 debonded sides, and 2 × 6 debonded sides. Figure 8 gives the stress distribution of MARC,VCFEM,ABAQUS at the horizontal and vertical centerline positions. As can be seen from the two figures, for each form of debonding, the stress plots computed by the two models are consistent, and the distribution of stress is different only at the interface between the inclusion and the matrix. The stress concentration in the hollow part is more pronounced in MARC and ABAQUS.

### Interfaical debonding of multiple hollow inclusions

**Figure 9:**
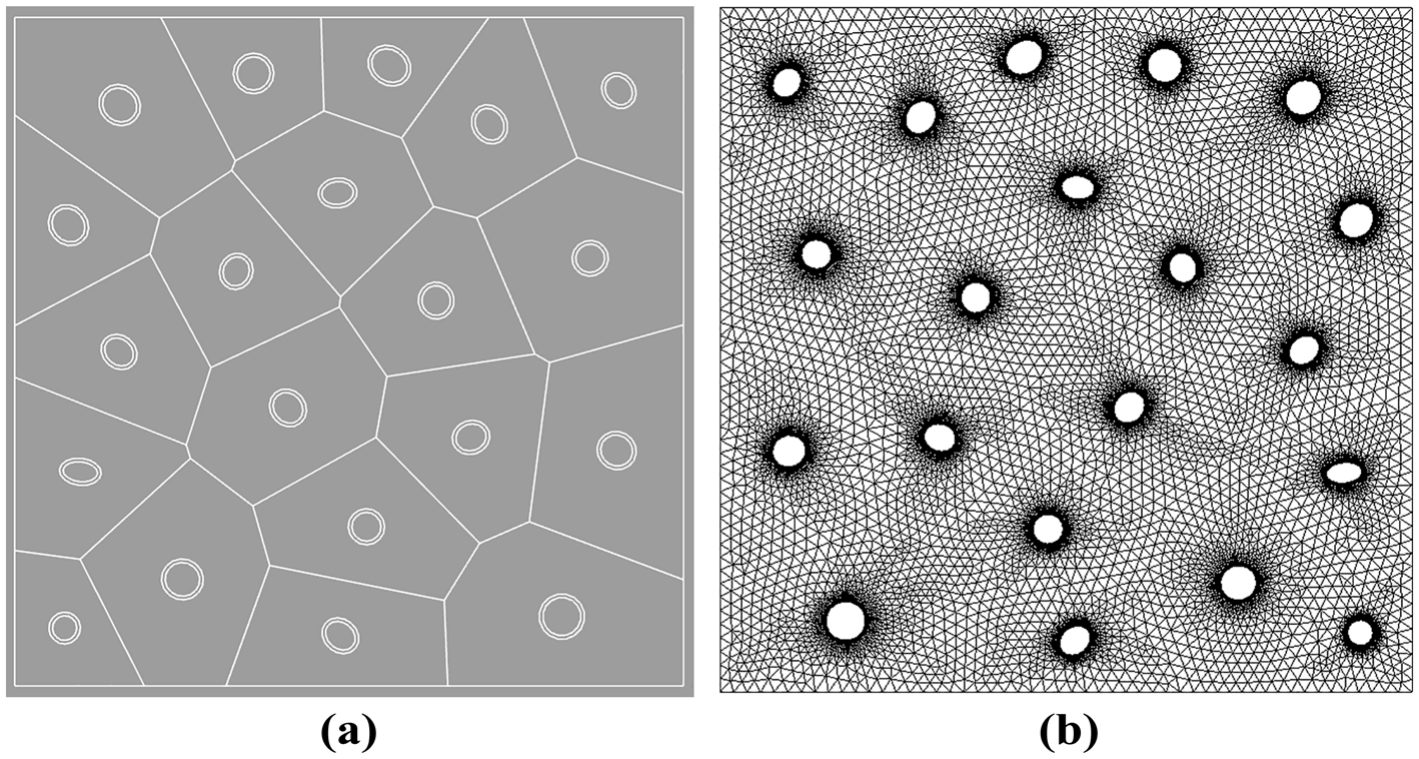
(**a**) VCFEM mesh structure model and (**b**) MARC mesh structure model.

In this calculation example, the main simulation is a complex hollow particle-reinforced composite, as shown in Fig. 9a, the target model has 20 randomly distributed elliptical hollow inclusions of arbitrary size and orientation on a square plate of $1 \times 1\;mm^{2}$. The 20 randomly distributed inclusions have a thickness of approximately 0.0055 mm, and the range of variation in the semi-major axis length of the hollow elliptical part is about 0.013 mm to 0.022 mm. The material properties of the model are the same as above, fixing the horizontal displacements at the left boundary, fixing the vertical displacements at the top and bottom boundaries, and applying loads at the right boundary.

Initially, all the inclusions interfaces were not debonding and each inclusion interface consisted of 16 straight edge segments. With the increase of load, the normal stresses at some interface nodes were larger than the given critical stresses, resulting in debonding of some interface segments, and the nodes with normal stresses larger than the given critical stresses were replaced by a node pair after remeshing. The stress functions for both the matrix and the inclusions within the elements of the VCFEM model contain 99 terms

The VCFEM model simulates the gradual damage generation of the entire structure under load as follows: a given displacement at the right end of the initial load is $0.0003\;mm$; for each subsequent incremental step, the displacement is incremented by $0.00001\;mm$, and the total number of load steps is 50. Critical normal stress at the interface was 8.05N.

**Figure 10:**
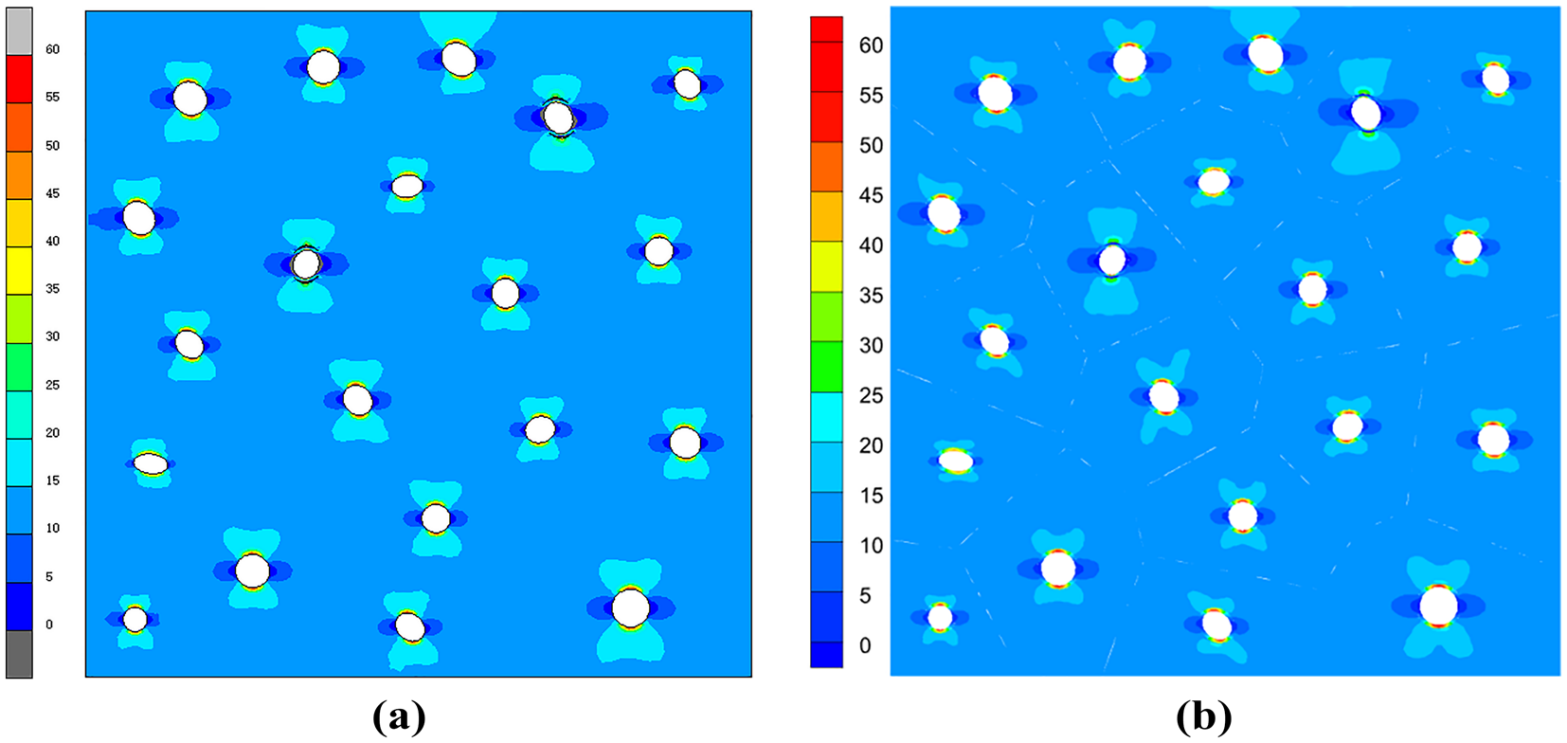
Comparison of horizontal stress cloud plots between (**a**) MARC model and (**b**) VCFEM model.

**Figure 11:**
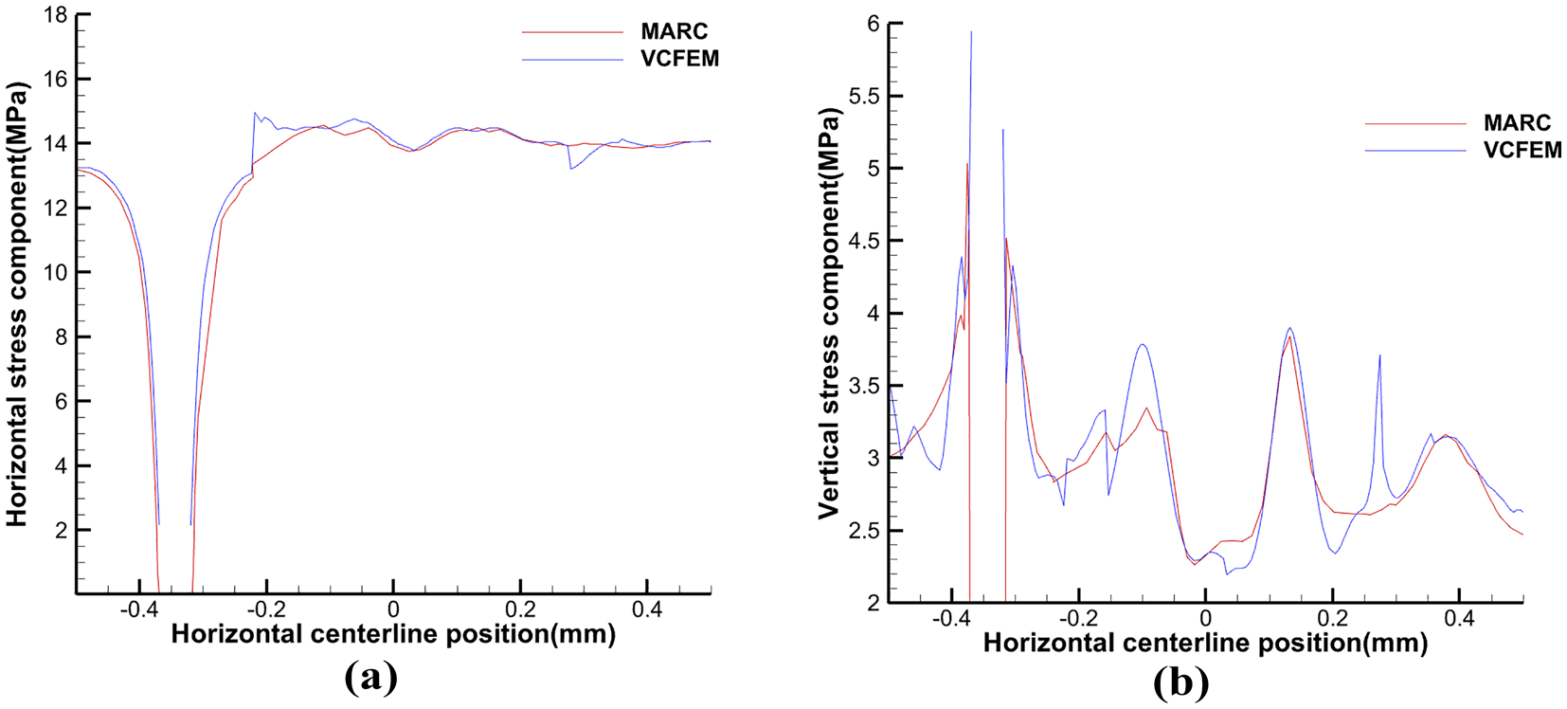
Distribution of stress (**a**) $\sigma_{x}^{{}}$, (**b**) $\sigma_{y}^{{}}$ on the horizontal centerline in MARC and VCFEM.

**Figure 12:**
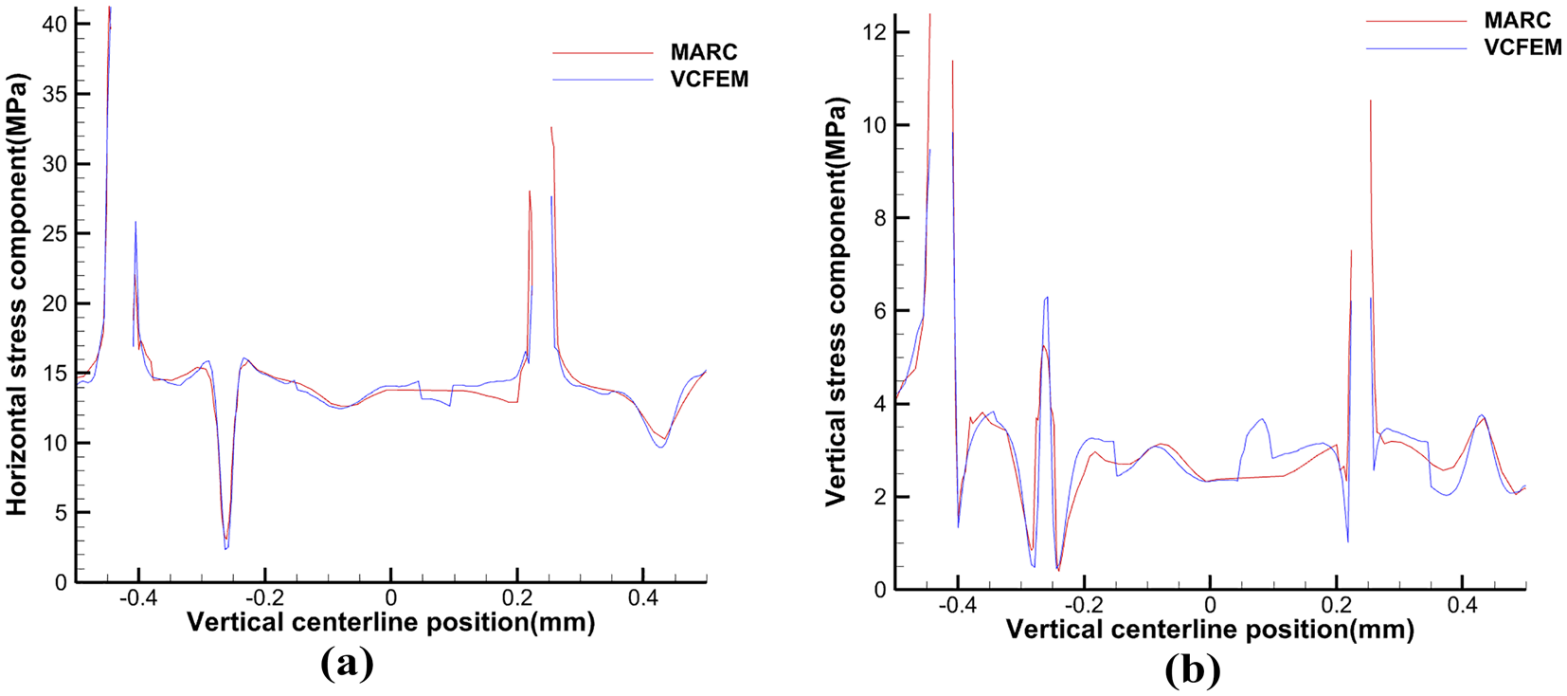
Distribution of stress (**a**) $\sigma_{x}^{{}}$, (**b**) $\sigma_{y}^{{}}$ on the vertical centerline in MARC and VCFEM.

In order to check the validity of the VCFEM, for the damage case obtained from the incremental step VCFEM calculation in step 4 (debonding produced at the ends of elements 5 and 16), a MARC model was built for the same damage case, as shown in Fig. 9b, the MARC model consists of 30,027 elements, which contain 15,974 nodes Fig. 10a,b. Stress $\sigma_{x}^{{}}$ cloud plots for MARC and VCFEM, respectively. Figures 11 and 12 show the stress distributions of the VCFEM model and MARC model on the horizontal and vertical centerlines. From these figures, it can be seen that the stress distributions of the two methods are in good agreement. The displacements of the internal nodes are related to the displacements of the external nodes of its own element, and the internal nodes are compressed within the element. Therefore, if no new cracks are created in the element, the calculation of the stiffness of the element is only needed in the first step of the whole calculation process.

**Figure 13:**
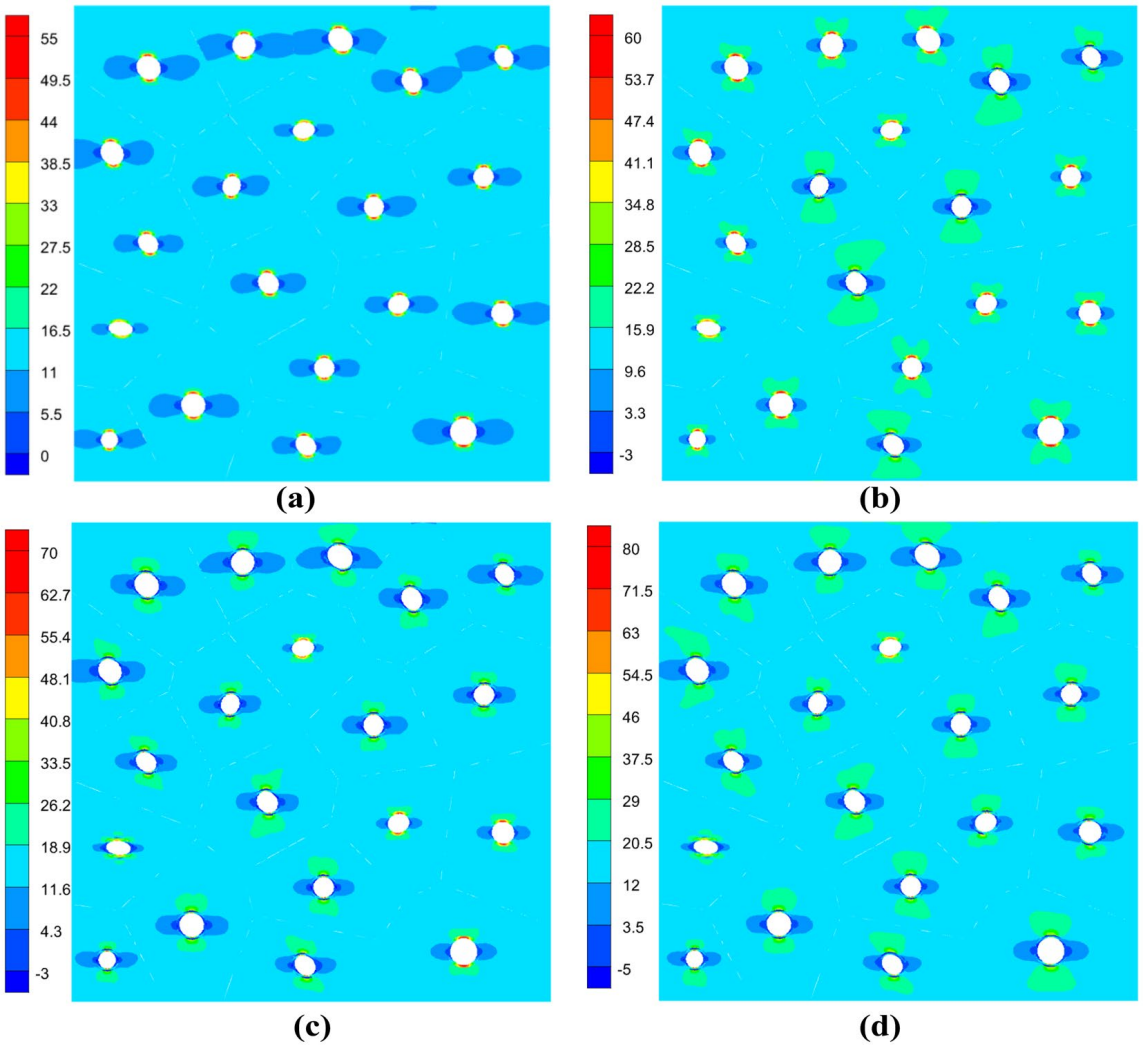
Horizontal stress cloud plots at (**a**) the 1st incremental step, (**b**) the 6th incremental step, (**c**) the 11th incremental step and (**d**) the 16th incremental step.

The results of the process simulation of the structural damage by the VCFEM model are as follows: the horizontal stresses at step 1 (0.00031 mm), step 6 (0.00036 mm), step 11 (0.00041 mm) and step 16 (0.00046 mm) are shown in Fig. 13. For step 1, the interface between the matrix and the hollow inclusions appears to be complete bonding. For step 6, six inclusions debonded at the interface. For step 11, fourteen inclusions experienced debonding at the interface. At step 16, all but one of the nineteen inclusions experienced debonding at the interface.

**Figure 14:**
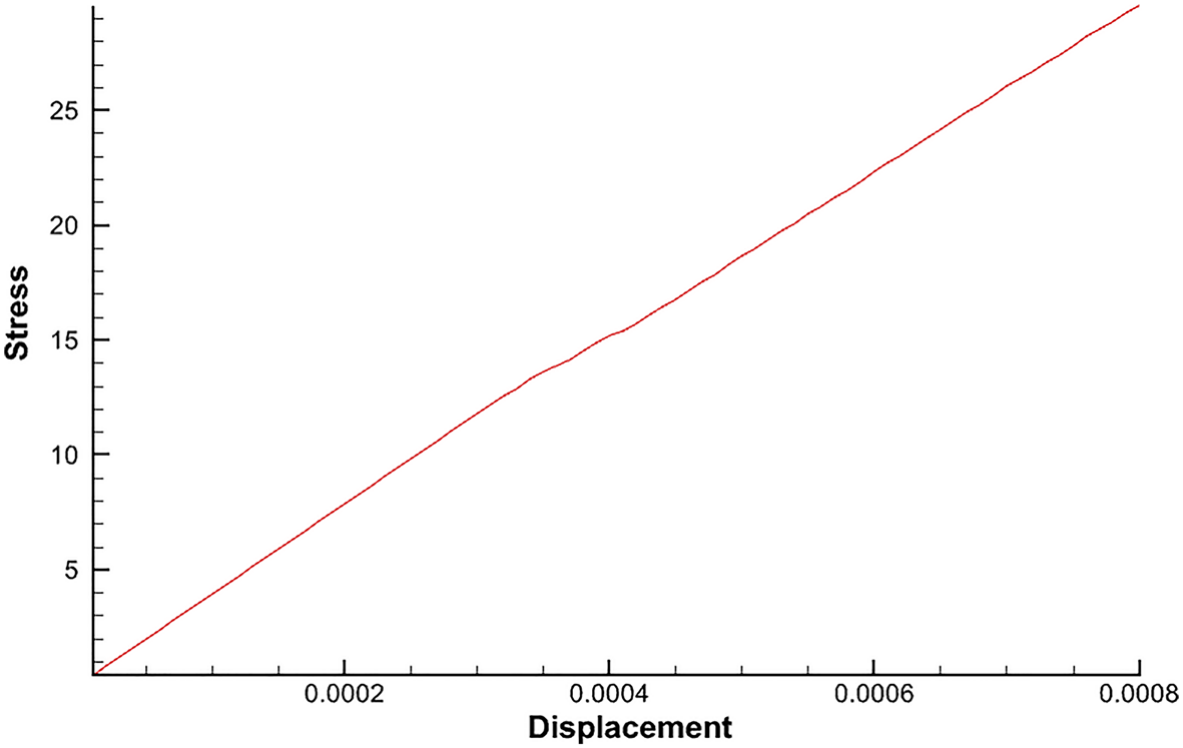
The variation of the right end constraint reaction force with the horizontal displacement of the right end during the process of damage generation.

In order to further understand the effect of interface debonding damage on the overall structure, the variation of the right end restraining force with the horizontal displacement of the right end is obtained, as shown in Fig. 14. In this case, the variation of the curve is roughly divided into three stages: (a) the initial linear stage, in which the displacement is less than 0.00033 mm, almost no debonding is generated and the restraining reaction force basically varies linearly with the displacement; (b) the massive damage stage, when the displacement of the right end is increased in the interval from 0.00033 mm to 0.00048 mm, there are subtle fluctuations in the increase in confining reaction force due to the production of large amounts of debonding. (c) Linear increase stage, when the displacement of the right end is greater than 0.00048mm, there still exists a small amount of delamination generation. The impact on the overall structure is very small and the restraining reaction force and displacement become linear change relationship again.

**Figure 15:**
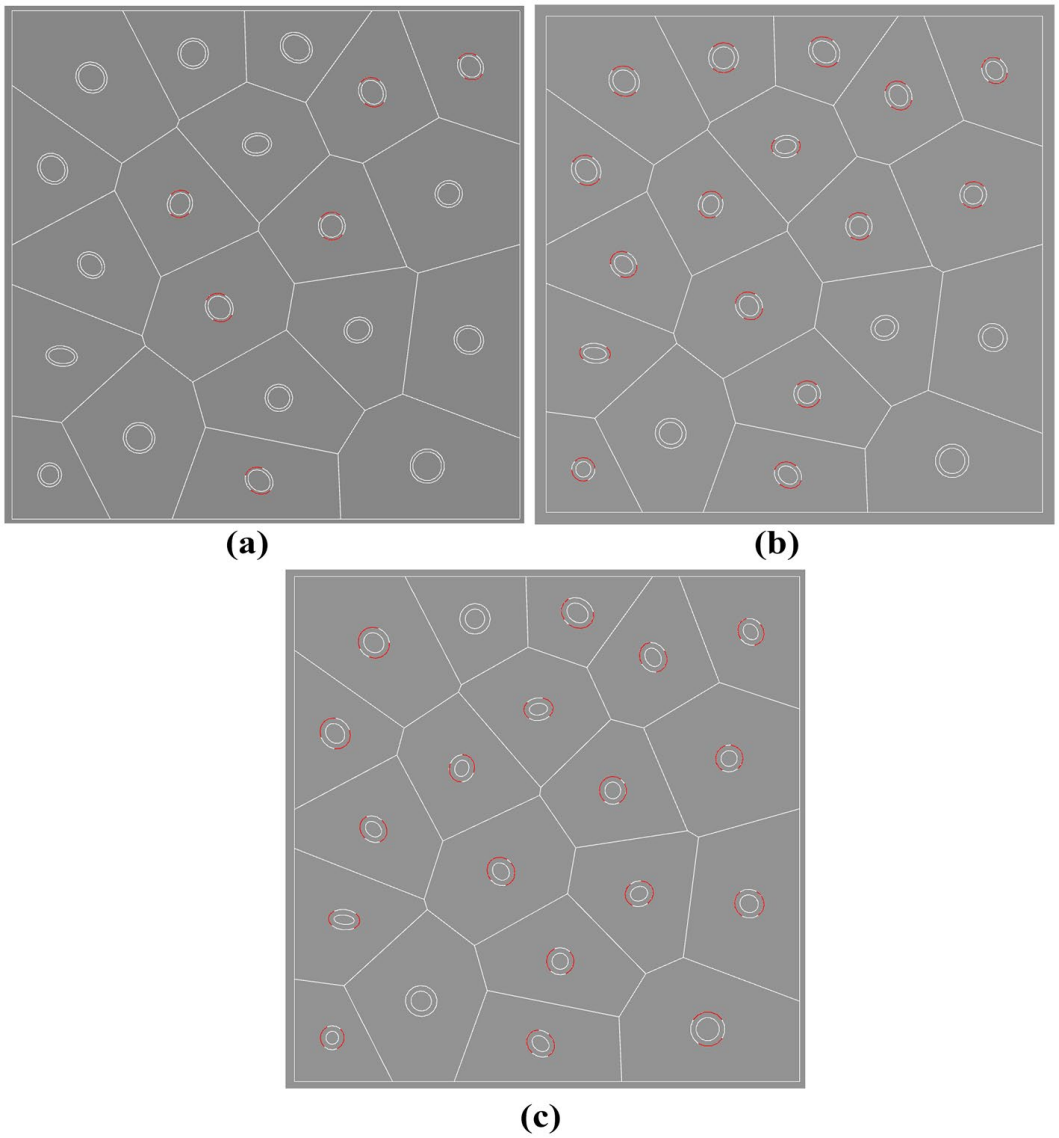
Damage profile of (**a**) 0.0055mm thickness, (**b**) 0.00825mm thickness and (**c**) 0.011 mm thickness in the sixth incremental step.

Figure 15 shows the debonding of elements with different inclusions wall thickness at the same sixth incremental step, Fig. 15a six inclusions debonding occurred, Fig. 15b sixteen inclusions debonding occurred, Fig. 15c eighteen inclusions debonding occurred. It can be seen that under the same displacement load, the larger the wall thickness of the inclusions in the hollow inclusion element the more prone to debonding damage.

## Conclusion

The new VCFEM was employed to model interface debonding of hollow particles reinforced composites. A remeshing strategy was developed to simulate the initiation and propagation of inclusion interface debonding. The efficacy and computational efficiency of this method were validated through comparisons with results obtained from established commercial software packages such as MARC and ABAQUS. Furthermore, this method was utilized to analyze the evolution of interface debonding damage in intricate composite structures.

The investigation also explored the impact of interface damage on horizontal displacement and constraint reaction forces, as well as the influence of hollow microsphere wall thickness on interface debonding. The findings underscore the effectiveness and computational efficiency of this approach in addressing complex engineering challenges.

## References

[1] Swetha, C. & Kumar, R. Quasi-static uni-axial compression behaviour of hollow glass microspheres/epoxy based syntactic foams. *Mater. Des.* **32**(8–9), 4152–4163 (2011). 10.1016/j.matdes.2011.04.058

[2] Scott, N. R. *et al.* Experimental and computational characterization of glass microsphere-cementitious composites. *Cem. Concr. Res.* **152**, 106671 (2022). 10.1016/j.cemconres.2021.106671

[3] Blanco, F. *et al.* Characteristics and properties of lightweight concrete manufactured with cenospheres. *Cem. Concr. Res.* **30**(11), 1715–1722 (2000). 10.1016/S0008-8846(00)00357-4

[4] Tiwari, V., Shukla, A. & Bose, A. Acoustic properties of cenosphere reinforced cement and asphalt concrete. *Appl. Acoust.* **65**(3), 263–275 (2004). 10.1016/j.apacoust.2003.09.002

[5] Hashin, Z. & Shtrikman, S. A variational approach to the theory of the elastic behaviour of multiphase materials. *J. Mech. Phys. Solids* **11**(2), 127–140 (1963). 10.1016/0022-5096(63)90060-7

[6] Mori, T. & Tanaka, K. Average stress in matrix and average elastic energy of materials with misfitting inclusions. *Acta Metallurgica* **21**(5), 571–574 (1973). 10.1016/0001-6160(73)90064-3

[7] Hill, R. A self-consistent mechanics of composite materials. *J. Mech. Phys. Solids* **13**(4), 213–222 (1965). 10.1016/0022-5096(65)90010-4

[8] Bardella, L. *et al.* A critical evaluation of micromechanical models for syntactic foams. *Mech. Mater.* **50**, 53–69 (2012). 10.1016/j.mechmat.2012.02.008

[9] Lee, K. J. & Westmann, R. A. Elastic properties of hollow-sphere-reinforced composites. *J. Compos. Mater.* **4**(2), 242–252 (1970). 10.1177/002199837000400209

[10] Huang, J. S. & Gibson, L. J. Elastic moduli of a composite of hollow spheres in a matrix. *J. Mech. Phys. Solids* **41**(1), 55–75 (1993). 10.1016/0022-5096(93)90063-L

[11] Porfiri, M. & Gupta, N. Effect of volume fraction and wall thickness on the elastic properties of hollow particle filled composites. *Compos. B Eng.* **40**(2), 166–173 (2009). 10.1016/j.compositesb.2008.09.002

[12] Yu, M. & Ma, Y. Effects of particle clustering on the tensile properties and failure mechanisms of hollow spheres filled syntactic foams: A numerical investigation by microstructure-based modeling. *Mater. Des.* **47**, 80–89 (2013). 10.1016/j.matdes.2012.12.004

[13] Weise, J. *et al.* Production and properties of 316 L stainless steel cellular materials and syntactic foams. *Steel Res. Int.* **85**(3), 486–497 (2014). 10.1002/srin.201300131

[14] Tagliavia, G., Porfiri, M. & Gupta, N. Analysis of flexural properties of hollow-particle filled composites. *Compos. B Eng.* **41**(1), 86–93 (2010). 10.1016/j.compositesb.2009.03.004

[15] Zhang, J. & Katsube, N. A hybrid finite element method for heterogeneous materials with randomly dispersed rigid inclusions. *Int. J. Numer. Methods Eng.* **38**(10), 1635–1653 (1995). 10.1002/nme.1620381004

[16] Ghosh, S. & Mukhopadhyay, S. N. A material based finite element analysis of heterogeneous media involving Dirichlet tessellations. *Comput. Methods Appl. Mech. Eng.* **104**(2), 211–247 (1993). 10.1016/0045-7825(93)90198-7

[17] Moorthy, S. & Ghosh, S. A model for analysis of arbitrary composite and porous microstructures with Voronoi cell finite elements. *Int. J. Numer. Methods Eng.* **39**(14), 2363–2398 (1996). 10.1002/(SICI)1097-0207(19960730)39:14<2363::AID-NME958>3.0.CO;2-D

[18] Moorthy, S. & Ghosh, S. Adaptivity and convergence in the Voronoi cell finite element model for analyzing heterogeneous materials. *Comput. Methods Appl. Mech. Eng.* **185**(1), 37–74 (2000). 10.1016/S0045-7825(99)00349-7

[19] Guo, R., Shi, H. & Yao, Z. Numerical simulation of thermo-mechanical fatigue properties for particulate reinforced composites. *Acta Mech. Sinica* **21**(2), 160–168 (2005). 10.1007/s10409-005-0024-z

[20] Zhang, R. & Guo, R. Multiphase hybrid stress finite element analysis of heterogeneous media by simple mesh: One element with one interface. *Int. J. Numer. Methods Eng.* **121**(12), 2767–2782 (2020). 10.1002/nme.6330

[21] Zhang, R. & Guo, R. Determination of crack tip stress intensity factors by singular Voronoi cell finite element model. *Eng. Fract. Mech.* **197**, 206–216 (2018). 10.1016/j.engfracmech.2018.04.042

[22] Ghosh, S. *et al.* Interfacial debonding analysis in multiple fiber reinforced composites. *Mech. Mater.* **32**(10), 561–591 (2000). 10.1016/S0167-6636(00)00030-2

[23] Li, S. & Ghosh, S. Extended Voronoi cell finite element model for multiple cohesive crack propagation in brittle materials. *Int. J. Numer. Methods Eng.* **65**(7), 1028–1067 (2006). 10.1002/nme.1472

[24] Li, S. & Ghosh, S. Multiple cohesive crack growth in brittle materials by the extended Voronoi cell finite element model. *Int. J. Fract.* **141**, 373–393 (2006). 10.1007/s10704-006-9000-2

[25] Zhang, R., Wang, T. & Guo, R. Modeling of interphases in multiple heterogeneities reinforced composites using Voronoi cell finite elements. *Acta Mech. Sin.* **36**, 887–901 (2020). 10.1007/s10409-020-00978-9

[26] Han, N. & Guo, R. Two new Voronoi cell finite element models for fracture simulation in porous material under inner pressure. *Eng. Fract. Mech.* **211**, 478–494 (2019). 10.1016/j.engfracmech.2019.01.012

[27] Hao, W. Y., Guo, R. & Han, N. A two-dimensional VCFEM formulated with plastic, creep and thermal strain for simulate fatigue of porous material. *Compos. Struct.* **252**, 112598 (2020). 10.1016/j.compstruct.2020.112598

[28] Rao, J., Guo, R. & Zhang, R. The numerical simulation of particulate reinforced composites by using a two-dimensional VCFEM formulated with plastic, thermal, and creep strain. *Compos. Struct.* **330**, 117825 (2024). 10.1016/j.compstruct.2023.117825

